# Summertime

**Published:** 2004-06-15

**Authors:** Lynne S. Wilcox

*Dandelion Wine* is a lyrical coming-of-age story set in Green Town, Illinois, in the early 20th century (1). Douglas Spaulding is 12 years old, and summertime has arrived. When it’s a new summer, you need new shoes. In fact, you need “Royal Crown Cream-Sponge Para Litefoot Tennis Shoes.” But Doug doesn’t have the money. He bargains with Mr. Sanderson, the proprietor of the local shoe store:

“Soon as I get those shoes on, you know what *happens*? . . . Bang! I deliver your packages, pick up packages, bring your coffee, burn your trash, run to the post office, telegraph office, library! You’ll see twelve of me in and out, in and out, every minute.”

Many readers older than 50 will recognize Doug. He may have a different name, he may want a bike instead of tennis shoes, he may like root beer instead of lemonade. Still, it’s summertime, and he’s in 12 places all at once.

That story took place more than 75 years ago. Today, too few U.S. children enjoy the magic of an active, outdoor summer. They may not even recognize the season, because most of their entertainment is indoors and sedentary. Children spend up to four-and-a-half hours each day in front of a television or computer screen (2). I recently made a field trip to a large toy store. The front of the store was filled with music videos, computer games, DVDs, and other entertainment technologies that require little physical effort. Where were the bicycles, skate boards, and soccer balls? Along the back wall of the store.

It seems unlikely that toy manufacturers and store managers are plotting to keep kids sedentary. They are simply following good market practice by displaying their most popular merchandise in prominent locations. Meanwhile, obesity among individuals aged six to 19 years has tripled since the 1960s (3).

The July issue of *Preventing Chronic Disease* includes two articles on VERB™, a multiethnic campaign to promote physical activity among *tweens*, or children aged nine to 13 years (4,5). Congress appropriated $125 million in 2001 to the Centers for Disease Control and Prevention to develop a national media campaign to change children’s health behaviors. The VERB campaign features general media spots for all children, special spots directed toward ethnic and racial groups, and parent materials to encourage children’s physical activity. Campaign planners use process and outcome evaluations to assess the effectiveness of this mass communications approach to changing the attitudes and behaviors of tweens and their parents (4).

The design and operation of the VERB campaign is a remarkable achievement. If the evaluation results show changes in children’s physical activity levels over time, the campaign will be an even more noteworthy accomplishment. Both the successes and challenges of developing and implementing such campaigns in the United States are discussed in a commentary in this issue (6). While VERB targets only children aged nine to 13, the campaign hopes to shape the attitudes and behaviors of these individuals as they age: marketers of other products for tweens have found that children who become consumers of a product often are loyal to those products into adulthood (7).

VERB offers public health lessons beyond the nuts and bolts of this campaign. First, to affect the health habits of a generation, address the cohort moving from childhood to early adolescence — when kids begin to make their own decisions and to experience influences beyond the home. Second, to create major changes in public attitudes, commit serious resources. VERB demonstrates that with proper financial investment, public health messages can attract wide attention. Third, know your audience. Time spent researching the interests of a special group can make the difference between an effective campaign and an ineffective one. Fourth, to create a credible public health campaign, include evaluation of results as an essential component.

These statements seem simple and self-evident, but many public health professionals have spent their careers in underfunded programs. Lack of resources may cause audience research and results evaluation to take a back seat to the overwhelming pressures of launching a new campaign. VERB provides an excellent example of how to use social marketing principles, which include research and evaluation, to design a successful national public health campaign.

Back in Green Town, Doug persuades Mr. Sanderson to try a pair of Litefoot tennis shoes:

[Sanderson] laced the tennis shoes to his long narrow feet. They looked detached and alien down there next to the dark cuffs of his business suit. . . . [H]e began to sink deep in the shoes, to flex his toes, limber his arches, test his ankles. He rocked softly, secretly, back and forth in a small breeze from the open door.

Then, Mr. Sanderson hands Doug a pair of Litefoot shoes and listens wistfully to the boy running away down the street.

*Dandelion Wine* was set during a time when adults put their tennis shoes away with their youth. Over the last decade, U.S. adults as well as children have demonstrated sharp rises in obesity. All of us can benefit from lacing up our tennis shoes and joining Doug and the kids of VERB in running away into summertime.
